# Legal strategies to improve physical activity in populations

**DOI:** 10.2471/BLT.20.273987

**Published:** 2021-05-04

**Authors:** Tracy Nau, Ben J Smith, Adrian Bauman, Bill Bellew

**Affiliations:** a The Australian Prevention Partnership Centre, Prevention Research Collaboration, University of Sydney, Charles Perkins Centre Level 6, John Hopkins Drive, Camperdown NSW 2006, Sydney, NSW, Australia.

## Abstract

The World Health Assembly has adopted the World Health Organization’s (WHO) recommended target of achieving a 15% reduction in physical inactivity by 2030. The WHO Global Action Plan on Physical Activity provides a framework for countries to achieve this, using a systems-based approach to address the social and environmental determinants of physical inactivity. Lack of progress in many countries indicates a need to identify new ways of addressing this public health priority. WHO continues to highlight the importance of legislative and regulatory measures within the multicomponent and multisectoral action needed to reduce physical inactivity. Yet research into the role of law for addressing physical inactivity has been limited, in contrast to the legal approaches to other major noncommunicable disease risk factors such as smoking and alcohol use. Conceptual frameworks for public health law offer a method for mapping and understanding the determinants, mechanisms and outcomes of law-making for the promotion of physical activity within populations. We describe the development and application of a framework that aligns legal strategies with the WHO Global Plan policy objectives. This new framework – the Regulatory Approaches to Movement, Physical Activity, Recreation, Transport and Sport – can help policy-makers to use the untapped potential of legal interventions to support or strengthen a whole-system response for promoting physical activity. The framework illustrates the role of legal interventions to improve physical activity and identifies opportunities for research to advance understanding, implementation and evaluation of legal responses to this issue.

## Résumé

L'Assemblée mondiale de la Santé a adopté l'objectif recommandé par l'Organisation mondiale de la Santé (OMS) visant à réduire l'inactivité physique de 15% d'ici 2030. L'OMS a élaboré un Plan d'action mondial pour l'activité physique, qui propose aux pays des orientations leur permettant d'atteindre cet objectif, grâce à une approche systémique qui aborde les déterminants sociaux et environnementaux à l'origine de l'inactivité physique. L'absence de progrès dans de nombreux pays indique un besoin d'identifier de nouveaux moyens de faire de cet enjeu de santé publique une priorité. L'OMS continue à souligner l'importance des mesures législatives et réglementaires au cœur de l'action multisectorielle à composantes multiples nécessaire à la promotion de l'exercice physique. Pourtant, les recherches sur la capacité de la loi à lutter contre l'inactivité physique sont limitées, contrairement aux démarches juridiques entamées vis-à-vis d'autres grands facteurs de risque de maladies non transmissibles, comme la consommation de tabac ou d'alcool. Les cadres théoriques régissant le droit sanitaire offrent des méthodes d'analyse et de compréhension des déterminants, mécanismes et impacts du travail législatif sur la promotion de l'activité physique au sein des populations. Dans cet article, nous décrivons le développement et l'application d'un cadre qui aligne les stratégies juridiques sur les objectifs politiques du Plan mondial de l'OMS. Ce cadre inédit – les réglementations relatives au mouvement, à l'activité physique, aux loisirs, au transport et au sport – peut aider les législateurs à utiliser le potentiel inexploité des interventions légales pour soutenir ou renforcer une réponse globale destinée à encourager l'exercice physique. Il illustre le rôle des interventions légales visant à améliorer l'activité physique et identifie les possibilités de recherche en vue de faire progresser la compréhension, la mise en œuvre et l'évaluation des solutions juridiques apportées à ce problème.

## Resumen

La Asamblea Mundial de la Salud ha adoptado el objetivo recomendado por la Organización Mundial de la Salud (OMS) de lograr una reducción del 15% de la inactividad física para 2030. El Plan de Acción Mundial de la OMS sobre la Actividad Física proporciona un marco para que los países lo logren, utilizando un enfoque basado en sistemas para abordar los determinantes sociales y ambientales de la inactividad física. La falta de progreso en muchos países indica la necesidad de identificar nuevas formas de abordar esta prioridad de salud pública. La OMS sigue destacando la importancia de las medidas legislativas y reglamentarias dentro de la acción multicomponente y multisectorial necesaria para reducir la inactividad física. Sin embargo, la investigación sobre el papel de la ley para abordar la inactividad física ha sido limitada, en contraste con los enfoques legales de otros factores de riesgo de enfermedades no transmisibles importantes, como el tabaquismo y el consumo de alcohol. Los marcos conceptuales del derecho de la salud pública ofrecen un método para trazar y comprender los determinantes, mecanismos y resultados de la elaboración de leyes para la promoción de la actividad física en las poblaciones. Describimos el desarrollo y la aplicación de un marco que alinea las estrategias legales con los objetivos políticos del Plan Global de la OMS. Este nuevo marco –enfoques normativos del movimiento, la actividad física, el ocio, el transporte y el deporte– puede ayudar a los responsables políticos a utilizar el potencial sin explotar de las intervenciones legales para apoyar o reforzar una respuesta de todo el sistema para promover la actividad física. El marco ilustra el papel de las intervenciones legales para mejorar la actividad física e identifica las oportunidades de investigación para avanzar en la comprensión, implementación y evaluación de las respuestas legales a este tema.

## ملخص

اعتمدت جمعية الصحة العالمية الهدف الذي أوصت به منظمة الصحة العالمية (WHO)، والذي يتمثل في تحقيق خفض بنسبة 15% في الخمول البدني بحلول عام 2030. إن خطة العمل العالمية لمنظمة الصحة العالمية بشأن النشاط البدني، توفر إطار عمل للدول لتحقيق هذا الهدف، وذلك باستخدام نهج قائم على النظم للتعامل مع المحددات الاجتماعية والبيئية للخمول البدني. يشير عدم إحراز تقدم في العديد من الدول إلى الحاجة إلى تحديد طرق جديدة للتعامل مع هذه الأولوية الصحية للعامة. تواصل منظمة الصحة العالمية التركيز على أهمية التدابير التشريعية والتنظيمية ضمن الإجراءات متعددة المكونات ومتعددة القطاعات، اللازمة للحد من الخمول البدني. ورغم ذلك، فإن الأبحاث الخاصة بدور القانون في التعامل مع الخمول البدني كانت محدودة، على العكس من أساليب التعامل القانونية مع عوامل خطر الأمراض غير المعدية الرئيسية الأخرى، مثل التدخين وتعاطي الكحول. توفر أطر العمل المفاهيمية لقانون الصحة العامة طريقة لتخطيط وفهم محددات وآليات ونتائج وضع القوانين الساعية للارتقاء بالنشاط البدني بين السكان. نحن نقوم بوصف تطوير وتطبيق إطار عمل يتولى التنسيق بين الاستراتيجيات القانونية وأهداف سياسة الخطة العالمية لمنظمة الصحة العالمية. إن إطار العمل الجديد هذا (وهو الأساليب التنظيمية للحركة والنشاط البدني والترفيه والنقل والرياضة)، يمكنه أن يساعد واضعي السياسات على استخدام الإمكانات غير المستغلة للتدخلات القانونية لدعم أو تقوية استجابة النظام بأكمله للارتقاء بالنشاط البدني. يوضح إطار العمل دور التدخلات القانونية لتحسين النشاط البدني، ويحدد فرص الأبحاث لتعزيز فهم وتنفيذ وتقييم الاستجابات القانونية لهذه القضية.

## 摘要

世界卫生大会通过了了世界卫生组织 (WHO) 建议的目标，即到 2030 年不参加体育活动的人数减少 15%。WHO《体育活动全球行动计划》为各国实现该目标提供了框架，以使用系统方法解决缺乏运动的社会和环境决定因素。多个国家没有取得进展表明需要找到解决该公共卫生重点问题的新方法。WHO 不断强调立法和监管措施在减少缺乏运动所需的多组分和多部门行动中的重要性。然而，与其它重大非传染性疾病风险因素（如吸烟和饮酒）的法律措施相比，关于法律在解决缺乏运动中作用的研究比较有限。公共卫生法律的概念性框架提供绘制和了解提高人口体育活动的立法决定因素、机制和成果的方法。我们描述了符合 WHO 全球计划政策目标法律策略的框架制定和应用过程。该新框架——运动、体育活动、娱乐、交通与体育活动监管办法——可帮助政策制定者使用法律干预的未开发潜力支持或加强促进体育活动的全系统响应。框架介绍了法律干预对促进体育活动的作用，并确定了提高对该问题法律响应的了解、实施和评估的研究机会。

## Резюме

Всемирная ассамблея здравоохранения приняла цель, рекомендованную Всемирной организацией здравоохранения (ВОЗ), — добиться сокращения физической неактивности среди населения на 15% к 2030 году. Глобальный план действий ВОЗ по повышению уровня физической активности обеспечивает странам основу для достижения этой цели с использованием системного подхода для определения социальных и экологических детерминант недостаточной физической активности. Отсутствие прогресса во многих странах указывает на необходимость определения новых способов решения этой приоритетной задачи общественного здравоохранения. ВОЗ по-прежнему подчеркивает важность законодательных и нормативных мер в рамках многокомпонентных и многосекторальных действий, необходимых для снижения уровня физической неактивности. Однако исследований роли закона в решении проблемы недостаточной физической активности проводилось ограниченное количество, в отличие от правовых подходов к другим основным факторам риска развития неинфекционных заболеваний, таким как курение и употребление алкоголя. Концептуальные основы законодательства в области общественного здравоохранения предлагают метод для картирования и понимания детерминант, механизмов и результатов законотворческого процесса, направленного на повышение уровня физической активности среди населения. Авторы описывают разработку и применение механизма, который приводит правовые стратегии в соответствие с концептуальными целями Глобального плана ВОЗ. Этот новый механизм, включающий подходы к регулированию передвижения, физической активности, отдыха, транспорта и спорта, может помочь представителям правительственных структур использовать нереализованный потенциал правовых действий для поддержки или усиления ответных мер на уровне всей системы, направленных на поощрение физической активности. Механизм иллюстрирует роль правовых действий для повышения уровня физической активности и определяет возможности для исследований, направленных на углубление понимания, реализации и оценки правовых мер реагирования на эту проблему.

## Introduction

Physical inactivity – activity below the recommended levels for population health – is a substantial contributor to chronic disease, comorbidity and premature death worldwide^1^ and is a life-course problem that tends to track from youth into adulthood.^2^ In low- and middle-income countries, rapid and poorly regulated urbanization and industrialization has led to physical inactivity becoming an important component of noncommunicable disease risk.^3^ Physical inactivity may not register as a political priority in these countries, however, due to the need to act on more acute public health issues.^3^ Some causes of physical inactivity, such as dependence on private cars and poor urban planning, can also exacerbate poor health indirectly through their contribution to climate change and air pollution.^4^^,^^5^

Member States of the World Health Organization (WHO) have committed to a 15% reduction in physical inactivity by 2030.^6^ The WHO Global Action Plan on Physical Activity^7^ was released in 2018 in response to concerns about the prevalence of insufficient physical activity around the world and inadequate global progress to address this. The Global Plan provides a systems-based framework for countries to achieve the 2030 target by promoting more activity in societies (social norms and attitudes), environments (spaces and places), people (programmes and opportunities) and systems (governance and policy enablers), using comprehensive and multisectoral strategies.^7^ Even so, the global prevalence of inactivity has remained largely unchanged since 2001,^8^^,^^9^ and many countries appear unlikely to achieve the target. To help identify new ways of addressing this issue, we propose a framework of legal strategies for increasing physical activity and provide examples of how these align with the Global Plan policy objectives. 

## Legal approaches

Several of the determinants of physical activity identified in the Global Plan are shaped and put into practice by law and legal processes. For example, road safety and traffic laws influence walking and cycling environments;^10^ planning and environment laws determine access to open space for active recreation;^11^ education laws may affect the quality and amount of teaching time for sport and physical education in schools.^12^ The United Nations Human Settlement Programme has highlighted the inadequate provision of legislative standards for public space as one of the reasons for the poor allocation of land to streets and public spaces in many low- and middle-income countries.^13^ Law can also make an important contribution to the Global Plan’s objective of active systems. For example, establishing and defining the mandate, functions and powers of government institutions can enable or hinder cross-governmental and interagency cooperation and coordination for physical activity promotion.^14^^,^^15^ A practical illustration of this is the 2009 law that established the Healthy Transportation Compact in Massachusetts State in the United States of America (USA). The Compact was an interagency committee within the Massachusetts Department of Transport tasked with developing healthy transportation policy and related administrative and procedural mechanisms for improving walking and cycling environments.^16^ This legislative intervention increased coordination and communication across agencies and departments, and led to an estimated 106% increase (from 0.39% to 0.8%) in commuters travelling by bicycle between 2005 and 2014 in a State population of approximately 7 million people.^16^

The WHO Global Action Plan on noncommunicable diseases highlighted the importance of legal strategies and arrangements for the prevention and control of noncommunicable disease risk factors such as physical inactivity.^17^ Since then, limited global progress on prevention of noncommunicable diseases has led to strong recommendations from the WHO independent high-level commission on noncommunicable diseases that governments employ their full legal powers and increase effective regulation to address physical inactivity and other noncommunicable disease risk factors.^18^ Law-making options to regulate tobacco, alcohol and unhealthy eating are generally well known and primarily relate to decreasing the marketing, availability and consumption of unhealthy products. To some extent, similar strategies may be needed for physical activity to limit the appeal and use of certain activities or products (such as use of private cars) and to regulate industry lobbying that may undermine government efforts to promote environmentally sustainable and active modes of transport. However, unlike regulation of tobacco, alcohol and unhealthy food, the primary focus of regulatory strategies for physical activity will be to promote this behaviour and increase the availability of places and spaces to enable higher levels of regular physical activity. Beyond targeting the broader determinants of physical activity, including through urban planning, land use and zoning laws (which for example, can improve the availability, quality and connectivity of pedestrian and cycling infrastructure),^19^ there has been limited investigation of what regulations should involve. The lack of progress is partly due to the difficulty in identifying relevant laws from the wide range of laws that could conceivably impact physical activity. In other cases, identifying relevant areas of law is relatively straightforward, but difficult to interpret and understand the complexity of the applicable legal regime. Another issue is the legal environment whereby laws may be made across different levels of government (federal, state and local) and branches (legislature, executive and judiciary).^20^ These issues create challenges for understanding how the current legal situation might impact the physical activity of the population and for identifying effective legal interventions that might improve physical activity.

The field of public health law seeks to address such challenges to promote the understanding, development and use of law as a tool for promoting health.^21^ Public health law research in the area of tobacco and alcohol control is well advanced. Examples include the comprehensive surveillance of laws and related social and epidemiological trends; investigations into the determinants of law-making and innovation; and evaluations of the effectiveness, implementation and enforcement of legal interventions.^22^^–^^28^ International law has been used to powerful effect in tobacco control; the widely adopted WHO Framework Convention on Tobacco Control provides a tool for driving and defending comprehensive regulatory approaches to tobacco control, resulting in measurable impacts on amount and prevalence of tobacco consumption.^23^^,^^24^ The field of public health law is also expanding into nutrition. This move follows increasingly urgent calls by WHO for governments to use their regulatory powers to restrict the production and marketing of unhealthy food and address obesity.^17^^,^^18^^,^^29^^,^^30^

While research on public health law for physical activity is underdeveloped, initial mapping and analysis work is underway. Researchers in Australia, for example, have developed an analysis grid to aid the systematic scanning of legal and regulatory opportunities for influencing physical activity environments across key sectors and at various levels of government.^31^ In Canada and the USA, progress in developing tools and methods for legal surveillance has enabled the comprehensive and systematic mapping of laws for physical activity in settings such as childcare, schools and outdoor walking trails.^32^^–^^35^ Legal epidemiology is being developed as a way to analyse associations between legal variables and behavioural, environmental and health outcomes. In the USA, the Classification of Laws Associated with School Students initiative^36^ has used legal epidemiology to assess associations between state laws governing physical education in schools and outcomes such as physical education time in schools,^37^ physical education class attendance^38^ and students’ physical activity levels.^12^

Despite these advances, legal interventions for physical activity are at an early stage of research. Advancing the agenda for physical activity law requires better understanding about how the concepts, frameworks and methods developed by researchers in public health law can be applied to physical activity. The logic model of public health law research, published in 2010, identifies potential focal points for research generally.^21^ We believe that a more tailored conceptual framework for physical activity will provide greater clarity and direction to guide a more comprehensive research and government policy agenda.

## Conceptual frameworks

Conceptual frameworks have been developed in other areas of public health to understand the role and influence of legislation. In 1978 a framework was proposed to support the development of legislative approaches to prevent primary, secondary and tertiary disease and injury in Switzerland based on active, semi-active and passive legal mechanisms.^39^ Physical activity was not specified, although road injury prevention was included. Several examples can be found for tobacco control, beginning with an early model published in 1991 which hypothesized that individual compliance with smoke-free laws results from interactions between environmental support, education and attitudes.^40^ In 2019, researchers proposed a model to map out where and how past and future regulatory action can affect the use of tobacco products.^41^ Development of models for tobacco control policy is a continuing process, demonstrated by recent calls for an additional model to support the framing, organization and implementation of smoke-free policies in low- and middle-income countries.^42^

Developing a comprehensive conceptual framework requires a sound understanding of the relevant variables and processes affecting the design, implementation and impact of laws in public health.^43^ However, as the examples in tobacco control have shown, legal frameworks can evolve to reflect new understanding or can be constructed to address emerging issues or gaps in understanding. In the case of physical activity, developing a preliminary framework that can be built on as further knowledge is gained would be valuable. For clarity, we have defined the key law-related terms used in this paper in Box 1.

Box 1 **Definitions of key law-related terms used in this article**

Laws: policies that create legally defined rights and obligations.

Legislation: a type of law that is enacted by government, that can be categorized in terms of primary or secondary legislation. Primary legislation (for example, acts of parliament) typically set out objectives and scope of legislation and its relationship to existing laws; identify an executive authority (such as a government department) that is responsible for the law’s implementation; and identify who and what is to be governed by the law, the procedures to be followed and the means of enforcement. The primary legislation may enable an executive authority to develop secondary legislation (such as regulations, ordinances, orders) to prescribe more detailed provisions and procedures to support the implementation of the primary legislation.

Legal interventions: the ways in which the government may exercise its legal authority, which may include enacting new laws, amending laws, activating regulatory powers, and repealing (abandoning) laws or particular legislative provisions that are ineffective or detrimental.

Legal mechanisms: the pathways that explain how a particular law or legal intervention operates to produce the effects observed.

Legal strategies: the tactics and approaches used by legislation to achieve particular objectives.

Sources: Burris et al. 2010;^21^ World Health Organization, 2021.^44^

## Framework development

Defining the ways in which law can be used to encourage physical activity would offer a useful basis for research. Areas that could be investigated include: the determinants of law-making for physical activity; the prevalence and distribution of legal strategies across different areas of action; the factors that influence effective implementation and enforcement of law for physical activity; and the effectiveness and mechanisms by which different legal strategies influence outcomes depending on the specific policy context. A framework for physical activity should therefore incorporate two main dimensions: (i) the distinct types of legal strategies, and (ii) the policy domains where these strategies can be applied to physical activity and contribute to a whole-system response to the issue.^14^

### Types of legal strategies

Various options exist to categorize legal strategies. A general classification has been proposed according to whether the strategies are interventional, infrastructural or incidental.^21^ In relation to physical activity, interventional strategies would be those that are intended to directly influence the outcomes or mediators of physical activity (for example, requirements for schools to provide minimum amounts of physical education). Infrastructural strategies would be those that establish powers, duties and institutions (for example, laws that establish an intersectoral agency to encourage active travel such as walking and cycling to get to or from places). Incidental strategies would be those that are not overtly aimed at addressing physical activity but have the effect of supporting physical activity (for example, land use zoning regulations that promote mixed-use planning create communities where residents can walk or cycle to access daily destinations such as shops and schools). Within those broad classifications, there are different legal strategies that could be used, for example by drawing on a previously published classification of legal tools and strategies for preventing obesity.^14^ Interventions could be based on information strategies that require governments to run public information campaigns promoting the benefits of physical activity, or on economic strategies that establish funding schemes for creating safe routes for children to walk or cycle to schools. There could also be prescriptive strategies that require health professionals to attain certain competencies in physical activity counselling to acquire or maintain professional registration.

The Policy, Location and Access in Community Environments framework developed by researchers at the School of Public Health, University of Alberta offers further possible legal strategies.^45^ This policy analysis framework classifies interventions according to the degree to which they intrude on individual autonomy: do nothing or monitor the situation; provide information; enable choice; guide choice by changing the default; guide choice through incentives; guide choice by disincentives; restrict choice; eliminate choice. The framework assigns legal strategies that affect the way governments act into a separate category, labelled Reorient government action.^45^ However, this single category does not account for the multiple ways in which laws can enable reorientation of government action, for example by clarifying or amending objectives, powers and functions, by diverting or creating new revenue streams and by establishing new governance structures that can facilitate cross-agency working.^14^^,^^19^ Whole-system approaches to promoting physical activity require major shifts to the way in which governments traditionally operate.^46^ Determining the ways in which the law might facilitate these shifts would be a helpful addition to the framework.

The food system crosswalk framework developed by the Healthy Food Policy Project in the USA identifies possible legal strategies to improve access to healthy food, and highlights the intersection of these strategies with different parts of the food system to achieve this goal.^47^ The framework’s classification of legal strategies provides similar coverage to the Policy, Location and Access in Community Environments framework, but has a stronger emphasis on legal strategies directed at organizations and governments rather than the individual (for example, the strategies include creating a fund). For this reason, we based our framework for physical activity law on the legal strategies delineated by the Healthy Food Policy Project. 

### Policy domains for action

The second dimension of a physical activity conceptual framework needs to reflect the policy areas where legal interventions may support and strengthen a whole-system approach for increasing population physical activity. The WHO Global Action Plan on Physical Activity^7^ sets out a framework of actions for countries to take to accelerate progress, based around four strategic objectives and 20 accompanying policy actions. The objectives aim to shift norms and attitudes (objective 1: active societies); create supportive environments for physical activity (objective 2: active environments); increase programmes and opportunities for physical activity (objective 3: active people); and strengthen the systems needed to implement effective and coordinated international, national and subnational action to increase physical activity (objective 4: active systems).^7^

Some of the actions in the Global Plan are already legal in nature and relate to the active environments objective: planning and transport regulations to redistribute urban space in favour of pedestrians, cyclists and public transport and to provide public open space (action 2.1.3); road safety legislation (action 2.3.1); and building design regulations to encourage and enable physical activity in and around schools, workplaces, sport and recreation and other facilities (action 2.5). Other objectives and actions in the Global Plan may also be amenable to legal strategies, but further understanding is needed to develop clear recommendations.

## Framework for physical activity law

**Fig. 1:**
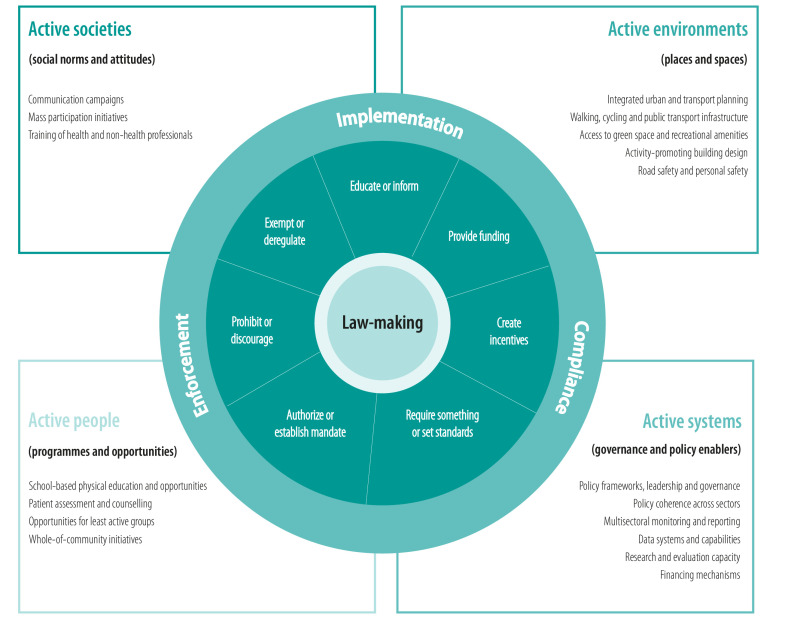
Regulatory Approaches to Movement, Physical Activity, Recreation, Transport and Sport framework

Note: The framework suggests a range of legal strategies that could be considered to address the action areas for physical activity in the WHO Global Action Plan on Physical Activity.^7^

The framework we propose in Fig. 1 directs attention of policy-makers and researchers to the range of ways in which legal strategies could be used or explored to address the WHO objectives for increasing population physical activity. We have adapted the logic model of public health law research^21^ to incorporate the two dimensions of physical activity described above. The framework outlines seven potential legal strategies (adapted from the Healthy Food Policy Project^47^) across four policy domains (based on the objectives in the Global Plan^7^). We have named our framework Regulatory Approaches to Movement, Physical Activity, Recreation, Transport and Sport (or RAMPARTS) to highlight the breadth of policy areas and types of physical activity that could be addressed using regulatory responses as part of a whole-system approach.

At the centre of our framework is law-making, which represents the multiple determinants that affect whether governments decide to pursue legal strategies to address physical activity (as opposed to other strategies that are not legally enforceable); whether proposed legislation is successfully enacted (or legislation that creates barriers to physical activity is successfully repealed); and the form and content of the final legislation. Any stage of law-making can be affected by industry which has long interfered with public health regulation in tobacco, alcohol and unhealthy food,^48^ but whose identity and influence are less clear for physical activity. Implementation, compliance and enforcement are highlighted in the outer ring of Fig. 1, as crucial factors influencing whether legal strategies achieve their intended objectives. We made some adaptations to the Healthy Food Policy Project classification^47^ to suit the promotion of physical activity as an outcome and to more comprehensively capture the ways in which the seven legal strategies we identify might apply to organizations and governments (Table 1). The table also provides examples of how each of the legal strategies could address the Global Plan^7^ objectives to promote physical activity. 

**Table 1:** Legal strategies to increase physical activity with examples mapped to policy actions of the WHO Global Action Plan on Physical Activity

Definition of proposed legal strategies	Example legal actions (WHO Global Plan actions)			
	Active societies	Active environments	Active people	Active systems
**Legal strategy 1: Awareness**				
Creates an educational or awareness campaign; provides information or teaches skills; creates information (e.g. performance indicators and routine surveillance data) or related reporting obligations (e.g. reporting this information to parliament)	Require transport agencies to promote public transport as an attractive alternative to car travel (Action 1.2). Require product warning labels on digital screen devices to identify potential harms of excessive use (Action NA)	Mandatory requirements for route signs to enhance pedestrian navigation and support active travel (Action 2.2). Require developers to prepare impact statements to enable assessment of planning proposals according to their likely effects on public transport, cycling and pedestrian networks, and on access to open green spaces (Action 2.1)	Require physical activity education to be provided as part of prenatal and antenatal care (Action 3.2)	Require periodic measurement and reporting of population physical activity including levels of use of public transport and parks (Action 4.2). Establish processes that enable public authorities to provide access to physical activity-related information and statistics held by them (Action 4.2). Require impact statements to support assessment of proposed laws according to their likely impact on community health and well-being (Action 4.1)
**Legal strategy 2: Funding**				
Creates a fund or allows a community to access an existing state or federal funding stream; prescribes a proportion of funding; or sets out funding or revenue conditions to improve access to physical activity	Establish cross-sectoral funding mechanisms for social marketing campaigns that promote physical activity and support the conduct of complementary community-based initiatives (Action 1.1, Action 1.2)	Make federal funding for transport conditional on state- and local-level improvements to walking or cycling infrastructure (Action 2.2). Direct revenue from car-parking levies and road tolls towards spending on public transport (Action 2.2). Establish a funding scheme for the construction of safe walking and cycling routes to schools (Action 2.3)	Make federal funding for education conditional on provision of minimum levels of physical education (Action 3.1). Allocate revenue from gambling or lotteries towards improving community-based sport and recreation opportunities (Action 3.3, Action 3.4, Action 3.5)	Establish funding criteria that prioritize the needs of the community and low socioeconomic areas (Action 4.5). Require the department of transport to prioritize the funding of transport projects that advance health and environment outcomes (Action 4.5)
**Legal strategy 3: Incentive**				
Creates an incentive to the adoption, change or maintenance of a particular practice or behaviour	Offer the general public tax deductions for work-related public transport expenses (Action NA). Establish a publicly-funded multidisciplinary health-care management scheme that allows inactive individuals with chronic disease or risk factors to access physiotherapy or other specialist support services to increase physical activity (Action NA)	Create incentives for property developers and building owners to adopt design standards that encourage people to be physically active in and around buildings (Action 2.5). Offer rebates on licence renewals for drivers with no traffic offences in the past 3 years (Action 2.3)	Create financial incentives for general medical practices to conduct routine assessment and counselling on physical activity (Action 3.2). Replace fringe benefits for provision of cars to employees, with fringe benefits for electric bicycles (Action 3.3)	Create incentives to encourage states and territories to improve population rates of physical activity (Action 4.5)
**Legal strategy 4: Standards**				
Requires something to be done or sets a standard	Amend accreditation schemes to require health professionals to complete continuing professional development and acquire competencies in physical activity counselling (Action 1.4)	Reduce vehicle speed limits to an enforceable 20 km per hour in local streets, park and playground zones and bicycle paths (Action 2.3). Establish or amend zoning and land use laws to require pedestrian-oriented development with convenient access to services (Action 2.1). Require schools to grant use of sports grounds and facilities to the community (Action 2.4)	Create a licensing scheme that requires early childhood services to meet or exceed specified quality standards such as for the provision of adequate and suitable areas and resources for active play (Action 3.1). Establish mandatory quality standards for the provision of physical activity opportunities in elderly people’s care homes (Action 3.4)	Require planning authorities to advance the population’s environmental, social and physical well-being when carrying out their functions (Action 4.1). Establish a bill of rights guaranteeing every person’s right to safe and healthy working conditions and reasonable limitation of working hours (Action 4.1)
**Legal strategy 5: Authorization**				
Expressly allows or establishes a mandate or authority for something in a way that supports or promotes physical activity	Confer functions on local councils to provide educational information to their communities about physical activity (Action 1.1, Action 1.2). Enable a government minister to issue codes of practice relating to the manner in which specified goods or services are advertised, sponsored or promoted for the purpose of reducing car dependency and physical inactivity (Action NA)	Confer powers on a government minister to authorize the closure of disused rail lines and their conversion to recreational routes (Action 2.4). Allow local governments to issue by-laws to reduce speed limits in their local council area (Action 2.3)	Specify that the objectives and functions of statutory sport bodies include improving equity of participation in organized sport (Action 3.3, Action 3.5)	Establish a new statutory body or mechanisms to improve interagency collaboration for physical activity (for example between transport, health and planning sectors) (Action 4.1). Amend the legislative mandate of statutory authorities to include physical activity-related objectives or functions (Action 4.1)
**Legal strategy 6: Prohibition**				
Prohibits or discourages a practice or behaviour (for example, by creating a disincentive)	Impose parking levies to discourage driving in or near city or town centres (Action NA)	Impose limits on the removal or destruction of green spaces or walking trails (Action 2.4). Prohibit motor vehicle access in certain zones to provide space for walking, cycling and active recreation (Action 2.2). Prohibit car parking and other obstruction in established cycleways (Action 2.2). Create traffic infringement penalties to deter vehicle speeding and aggressive behaviour towards pedestrians and cyclists (Action 2.3)	Prohibit the exclusion of any person from organized sport or recreation opportunities on the basis of gender, cultural or ethnic background, or disability (Action 3.3, Action 3.5)	NA
**Legal strategy 7: Exemption**				
Creates an exemption or deregulates something or someone in a way that supports or promotes physical activity	NA	Create exemptions from liability for schools that provide community access to sport and recreation grounds and facilities (Action 2.4). Exempt bicycles from restrictions on being carried on public transport (Action 2.2)	Exempt sport and recreational providers from liability where they have exercised duty of care to prevent injury (Action 3.3)	Create privacy law exemptions to allow non-government entities to disclose physical activity data to government agencies (Action 4.2)

NA: not applicable; WHO: World Health Organization.

Notes: We adapted legal strategies from the Healthy Food Policy Project’s classification of legal strategies for improving the food system.^47^ We based the examples on a combination of real-world and hypothetical examples which have been included for illustrative purposes only, independent of their effectiveness or suitability for any particular context. The action numbers in parentheses identify the WHO Global Action Plan on Physical Activity action to which the example relates.^7^ Some examples under the Active societies column do not have a WHO Global Plan action listed but we consider them relevant to the objective of shifting norms and attitudes.

Our framework does not prescribe certain legal strategies (or the use of law as a policy tool more generally) as appropriate or effective to promote any specific WHO objective or action area. The strategies to use would be determined by the local country context, the feasibility of those strategies within their existing legal frameworks and the country’s capacity for implementation and enforcement. However, we believe our framework can help identify the range of potential legal strategies and evidence gaps that may need to be addressed to guide the use of legal strategies in different contexts.

## Research and practice

As recognized in the WHO Global Action Plan on Physical Activity,^7^ each country will be at different stages of progress in addressing physical inactivity. While the 20 recommended policy actions in the Global Plan are universally applicable, the prioritization, feasibility and speed of implementation will vary according to context. The Global Plan therefore recommends that countries assess their current situation to ascertain existing areas of progress that could be strengthened and to identify policy opportunities and practice gaps. Low- and middle-income countries are increasingly recognizing the role of physical inactivity as a risk factor for noncommunicable diseases and are starting to assess the required infrastructure, strategies and resources to address this.^3^ Progress will require strong leadership and clear solutions initiated through government action and implementation of national policies and plans for promoting physical activity.^3^ Governments are uniquely empowered to legislate and regulate. The framework we propose may promote and guide discussion among policy-makers about where legal intervention could strengthen or support whole-system action for physical activity, and the resources required for implementation and enforcement of laws. Our framework also encourages broad consideration of regulatory approaches, beyond the prescriptive command-and-control type strategies that are traditionally associated with regulation. Such strategies may be effective and efficient to employ in resource-constrained environments.^49^

An initial step may involve legal mapping to understand the existing legal context across a spectrum of regulation types (from self-regulation to mandatory government regulation) that influence a particular Global Plan action area at a national or subnational level. Our framework and examples in Table 1 can assist the process of legal mapping, by identifying legal strategies for physical activity and encouraging a broad consideration of the types of policy areas where such strategies may be found.

After relevant laws have been identified, implementation assessment (usually using qualitative methods) could examine how and to what extent the law, as written, is implemented and enforced in practice.^21^ This process will show how existing law works, how decision-makers behave as a direct or indirect response to legal requirements, and how those decisions affect physical activity environments and behaviours.^50^ This assessment can generate plausible theoretical models of legal mechanisms for physical activity that can be tested and validated through systematic evaluation.

Both legal mapping and implementation studies may identify gaps and opportunities where laws can be developed or improved. In addition, studies analysing policy-making for physical activity can help identify and understand the factors influencing the successful enactment and implementation of legal interventions and to develop strategies to support this.^21^ Policy surveillance (a systematic type of legal mapping) will generate scientifically sound data to evaluate physical activity laws over time, to contribute to the evidence base and to inform future policy action. By building the evidence base, such research can strengthen political and public support for the enactment of laws relevant to physical activity.^19^^,^^21^ There are opportunities for further development of our proposed framework, or for modelling particular components of the framework, using the methods described above. The aim would be to integrate new knowledge and understanding about the variables that influence the making, implementation and enforcement of laws, and the effectiveness of legal strategies for enabling more physical activity. Research would be particularly important in low- and middle-income countries where local evidence is more limited. By strengthening the conceptual and practical linkages between law and physical activity, we can improve the value of the framework for informing a research agenda for law and physical activity and improving policy and practice.
